# Open peer review?

**DOI:** 10.1590/1518-8345.0000.4909

**Published:** 2026-04-27

**Authors:** Maíra Terra Garcia, Sigmar de Mello Rode

**Affiliations:** 1 Universidade Estadual Paulista, Institute of Science and Technology, Department of Biosciences and Oral Diagnosis, São José dos Campos, SP, Brazil.; 2 Universidade Estadual Paulista, Institute of Science and Technology, Department of Dental Materials and Prosthesis, São José dos Campos, SP, Brazil.

**Figure:**
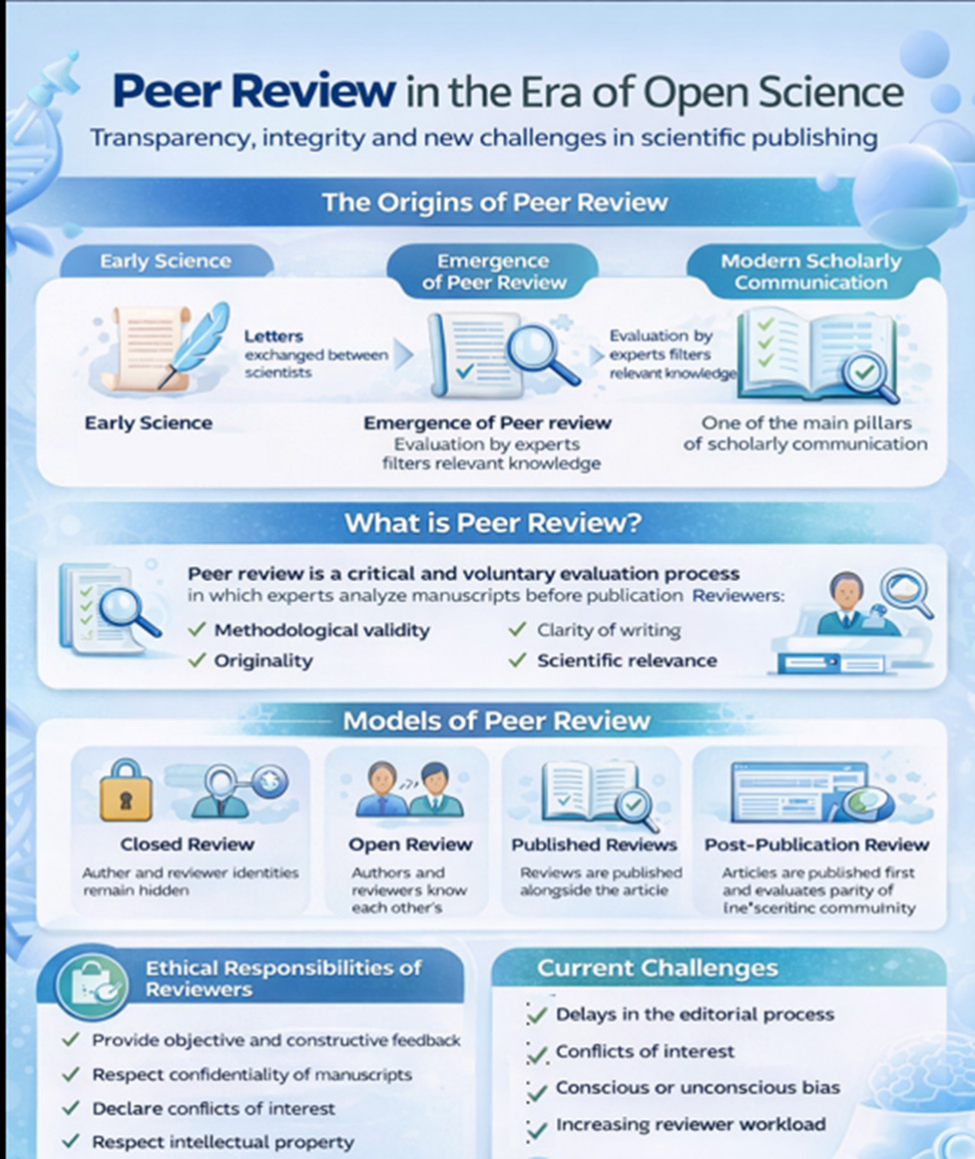

**Figure:**
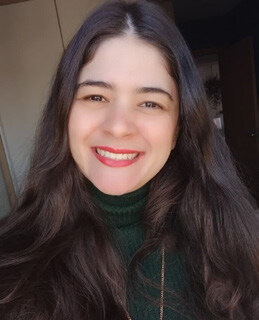

**Figure:**
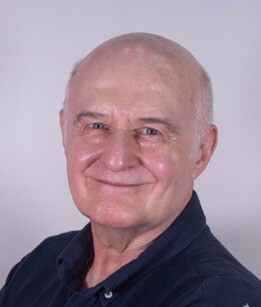

Initially, scientific articles consisted of communications and exchanges of letters between scientists. Due to the great diversity and presence of non-relevant information, a process of arbitration for these communications was eventually created, giving rise to what is now known as peer review.

Peer review can be defined as a process in which experts in a given field critically and voluntarily evaluate manuscripts submitted for publication, assessing their methodological validity, originality, clarity, and the relevance of their findings-ranging from fully closed to fully open formats. “Fully open reviews, in which the identities of authors and reviewers are known to both; open reviews published at the end of the article, creating space for post-publication discussions; and the replacement of traditional peer review with post-publication review are among the alternatives that have gained prominence as ways to evolve the original peer review process”^1^. In addition, peer review is considered one of the central pillars of scholarly communication. Although different modalities exist, they all share the same fundamental principle: ensuring integrity, rigor, and reliability in scientific output^2^. 

In the post-publication review model, manuscripts undergo basic technical screening (covering plagiarism, ethics, and formatting) and are immediately published on specific platforms (such as F1000Research). Critical evaluation by the scientific community occurs transparently on the article’s page, allowing authors to issue updated versions based on the feedback received. The primary challenge lies in engagement; without the editorial pressure of traditional journals, many articles may fail to receive comments, creating an “evaluation void” for less prominent works.

COPE^3^ provides some guidance for reviewers:

“Peer reviewers have an important role in ensuring the integrity of the scholarly record, but too often come to the role without any guidance and unaware of their ethical obligations.

Reviewers are expected to provide an unbiased, constructive critique of the manuscript. Reviews should be objective and constructive, ensuring feedback is clear and helpful to authors.

Reviewers should respect the confidentiality of the manuscripts they evaluate. They should not disclose any information about the work or use it for personal advantage, ensuring the integrity of the review process.

Reviewers should declare any potential conflicts of interest. This includes financial, personal relationships, or academic rivalries that could affect impartiality.

Reviewers are encouraged to complete their evaluations promptly to facilitate timely publication processes. Delays can hinder the dissemination of important research findings and affect authors’ careers.

The ethical obligations of reviewers include avoiding bias, respecting intellectual property rights, and recognising the contributions of others. Reviewers must uphold the highest ethical standards in their evaluations”. 

Reviewers are responsible for examining the accuracy, clarity, and significance of a manuscript, acting as true “gatekeepers” in the dissemination of scientific knowledge^4^. 

Since transparency is the basis of the integrity of the evaluation process, it should be open, so that it is possible to identify all actors and all communication in the process. There are several ways to open the process.

SciELO (Scientific Electronic Library Online)^5^ recommends that peer review of manuscripts should be as informed as possible. Articles must contain in the final version at least the name of the editor(s) responsible for the evaluation process. Journals should also offer reviewers and authors the option of opening their respective identities in order to favor interaction in the manuscript evaluation process. 

However, there are recurring criticisms of the peer review process, including delays in the editorial cycle, conflicts of interest, conscious or unconscious biases, and overwork for reviewers. The excessive burden on reviewers is a widely recognized reality, experienced reviewers are often requested repeatedly, and yet they accept these voluntary tasks because they understand that, without their continued participation, there would be no more peer-reviewed journals, which would delay the progress of science.

In this quest to optimize time, and to successfully achieve all the demands imposed by science, many reviewers are using artificial intelligence (AI) for peer review. At first, its use was presented as a technology capable of supporting editors and reviewers in a timely manner, but its use requires caution and critical reflection, as they bring important challenges. One of the main risks is the potential to reinforce biases already present in scientific literature, as these systems are trained with, can reproduce inequalities and discriminatory patterns of gender, ethnicity, institution of origin or writing style. Another problem lies in AI’s inability to evaluate theoretical, argumentative, and epistemological subtleties. While a human reviewer considers context, originality, and scientific relevance, AI tends to operate based on formal standards, without picking up on conceptual nuances or disruptive advances. This can result in unfair evaluations, hindering the development of new perspectives^6^. In addition, AI models often function as “black boxes”, without transparency about the criteria used for their recommendations, something incompatible with the need for clarity in the scientific editorial process.

It is very important that the journal has clear rules in its editorial policy about whether or not to allow the use of AI for both authors and reviewers, and that they must be explicit about how and which AI they used in the process. The generated product must always be curated by humans to correct any flaws, which is fundamental.

Peer review continues to be a dilemma for editors and authors. The more open the process, the greater the transparency and greater dissemination of knowledge, because the exchange of communication between author, reviewer, and editor will allow a greater understanding of the knowledge generated. 

One of the criticisms of the system is possible harassment, bullying (intimidation) and boycott (exclusion) for the disclosure of names, however, even anonymous evaluation is not a guarantee of protection.

On the contrary, open peer review enables a greater network with the interaction between the reviewer and the academy, in addition to attributing the proper credit for authorship to the reviewer while creating a bank of reviews that can serve as guidance to other reviewers, especially beginners.

Don’t be the last to join.
