# Supplementary material for: Open peer review?
Source: Rev Lat Am Enfermagem. 2026 Apr 27;34:e4909. doi: 10.1590/1518-8345.0000.4909 (PMC13124039; doi:10.1590/1518-8345.0000.4909)
Supplement: Translation

# Translation: Avaliação por pares aberta?

**DOI:** 10.1590/1518-8345.0000.4910

**Authors:** Maíra Terra Garcia, Sigmar de Mello Rode

**Affiliations:** 1 Universidade Estadual Paulista, Institute of Science and Technology, Department of Biosciences and Oral Diagnosis, São José dos Campos, SP, Brasil.; 2 Universidade Estadual Paulista, Institute of Science and Technology, Department of Dental Materials and Prosthesis, São José dos Campos, SP, Brasil.

Inicialmente, os artigos científicos consistiam em comunicações e trocas de cartas entre cientistas. Devido à grande diversidade e à presença de informações irrelevantes, acabou por ser criado um processo de arbitragem para essas comunicações, dando origem ao que hoje é conhecido como avaliação por pares. A avaliação por pares pode ser definida como um processo no qual especialistas em um determinado campo avaliam de forma crítica e voluntária os manuscritos submetidos para publicação, examinando sua validade metodológica, originalidade, clareza e a relevância de suas descobertas - variando de formatos totalmente fechados a totalmente abertos. “Avaliações totalmente abertas, em que a identidade de autores e avaliadores é conhecida por ambos; avaliações abertas publicadas ao final do artigo, abrindo espaço para discussões pós-publicação; e a substituição da avaliação por pares por avaliação pós-publicação estão entre as alternativas que ganharam destaque como formas da evolução do processo original de *peer review*.”^1^. Além disso, a avaliação por pares é considerada um dos pilares centrais da comunicação acadêmica. Embora existam diferentes modalidades, todas compartilham o mesmo princípio fundamental: garantir a integridade, o rigor e a confiabilidade da produção científica^2^.

No modelo de avaliação pós-publicação, os manuscritos passam por uma triagem técnica básica (abrangendo plágio, ética e formatação) e são imediatamente publicados em plataformas específicas (como F1000Research). A avaliação crítica pela comunidade científica ocorre de forma transparente na página do artigo, permitindo que os autores publiquem versões atualizadas com base no *feedback* recebido. O principal desafio reside no envolvimento; sem a pressão editorial das revistas tradicionais, muitos artigos podem não receber comentários, criando um “vazio de avaliação” para trabalhos menos proeminentes. 

O *Committee on Publication Ethics* (COPE)^3^ fornece algumas orientações para os avaliadores (tradução livre):

“Os avaliadores têm um papel importante em garantir a integridade do registro acadêmico, mas muitas vezes assumem essa função sem qualquer orientação e sem consciência de suas obrigações éticas. 

Espera-se que os avaliadores forneçam uma crítica imparcial e construtiva do manuscrito. As avaliações devem ser objetivas e construtivas, garantindo que o *feedback* seja claro e útil para os autores.

Os avaliadores devem respeitar a confidencialidade dos manuscritos que avaliam. Eles não devem divulgar nenhuma informação sobre o trabalho ou usá-la para vantagem pessoal, garantindo a integridade do processo de avaliação.

Os avaliadores devem declarar quaisquer conflitos de interesse potenciais. Isso inclui relações financeiras, pessoais ou rivalidades acadêmicas que possam afetar a imparcialidade.

Os avaliadores são incentivados a concluir suas avaliações prontamente para facilitar os processos de publicação em tempo hábil. Atrasos podem impedir a divulgação de importantes resultados de pesquisas e afetar a carreira dos autores.

As obrigações éticas dos avaliadores incluem evitar vieses, respeitar os direitos de propriedade intelectual e reconhecer as contribuições de terceiros. Os avaliadores devem manter os mais elevados padrões éticos em suas avaliações.”

Os avaliadores são responsáveis por examinar a precisão, clareza e importância de um manuscrito, atuando como verdadeiros “guardiões” na disseminação do conhecimento científico^4^. 

Como a transparência é a base da integridade do processo de avaliação, ele deve ser aberto, de modo que seja possível identificar todos os atores envolvidos e todas as comunicações no processo. Existem várias maneiras de abri-lo.

O SciELO (*Scientific Electronic Library Online*)^5^ recomenda que a avaliação por pares dos manuscritos seja o mais informada possível. Os artigos devem conter em sua versão final pelo menos o nome do(s) editor(es) responsável(is) pelo processo de avaliação. Os periódicos também devem oferecer aos avaliadores e autores a opção de divulgar suas respectivas identidades, a fim de favorecer a interação no processo de avaliação do manuscrito.

No entanto, há críticas recorrentes ao processo de avaliação por pares, incluindo atrasos no ciclo editorial, conflitos de interesse, vieses conscientes ou inconscientes e excesso de trabalho para os avaliadores. A carga excessiva sobre os avaliadores é uma realidade amplamente reconhecida: avaliadores experientes são solicitados repetidamente e, ainda assim, aceitam essas tarefas voluntárias porque entendem que, sem sua participação contínua, não haveria mais periódicos avaliados por pares, o que atrasaria o progresso da ciência.

Nessa busca por otimizar o tempo e atender com sucesso a todas as demandas impostas pela ciência, muitos avaliadores estão usando inteligência artificial (IA) para a avaliação por pares. Inicialmente, seu uso foi apresentado como uma tecnologia capaz de apoiar editores e avaliadores de maneira oportuna, mas seu uso requer cautela e reflexão crítica, pois traz desafios importantes. Um dos principais riscos é o potencial de reforçar vieses já presentes na literatura científica, pois esses sistemas são treinados com dados que podem reproduzir desigualdades e padrões discriminatórios de gênero, etnia, instituição de origem ou estilo de redação. Outro problema reside na incapacidade da IA de avaliar sutilezas teóricas, argumentativas e epistemológicas. Enquanto um avaliador humano considera o contexto, a originalidade e a relevância científica, a IA tende a operar com base em padrões formais, sem captar nuances conceituais ou avanços disruptivos. Isso pode resultar em avaliações injustas, dificultando o desenvolvimento de novas perspectivas^6^. Além disso, os modelos de IA muitas vezes funcionam como “caixas pretas”, sem transparência sobre os critérios utilizados para suas recomendações, algo incompatível com a necessidade de clareza no processo editorial científico.

É muito importante que o periódico tenha regras claras em sua política editorial sobre permitir ou não o uso de IA tanto para autores quanto para avaliadores, e que eles sejam explícitos sobre qual IA utilizaram no processo, e como. O produto gerado deve sempre a curadoria de humanos para corrigir quaisquer falhas, o que é fundamental.

A avaliação por pares continua sendo um dilema para editores e autores. Quanto mais aberto for o processo, maior será a transparência e maior será a disseminação do conhecimento, pois a troca de comunicação entre autor, avaliador e editor permitirá uma maior compreensão do conhecimento gerado.

Uma das críticas ao sistema é a possibilidade de assédio, *bullying* (intimidação) e boicote (exclusão) em função da divulgação de nomes, porém, mesmo a avaliação anônima não é garantia de proteção.

Pelo contrário, a avaliação por pares aberta permite maior rede de interação entre o avaliador e a academia, além de atribuir o devido crédito pela autoria ao avaliador, criando um banco de avaliações que pode servir de orientação para outros avaliadores, especialmente iniciantes.

Não seja o último a aderir.

# Translation: ¿Revisión por pares abierta?

**DOI:** 10.1590/1518-8345.0000.4908

**Authors:** Maíra Terra Garcia, Sigmar de Mello Rode

**Affiliations:** 1 Universidade Estadual Paulista, Institute of Science and Technology, Department of Biosciences and Oral Diagnosis, São José dos Campos, SP, Brasil.; 2 Universidade Estadual Paulista, Institute of Science and Technology, Department of Dental Materials and Prosthesis, São José dos Campos, SP, Brasil.

Inicialmente, los artículos científicos consistían en comunicaciones e intercambios de cartas entre científicos. Debido a la gran diversidad y a la presencia de información irrelevante, se creó un proceso de arbitraje para estas comunicaciones, dando lugar a lo que hoy se conoce como revisión por pares.

La revisión por pares puede definirse como un proceso en el que especialistas en un campo determinado evalúan de forma crítica y voluntaria los manuscritos presentados para su publicación, examinando su validez metodológica, originalidad, claridad y la relevancia de sus descubrimientos, y que puede variar desde formatos totalmente cerrados hasta totalmente abiertos. “Las revisiones totalmente abiertas, en las que la identidad de los autores y revisores es conocida por ambos; las revisiones abiertas publicadas al final del artículo, que dan lugar a debates posteriores a la publicación; y la sustitución de la revisión por pares por la revisión posterior a la publicación se encuentran entre las alternativas que han cobrado importancia como formas de evolución del proceso original de revisión por pares”^1^. Además, la revisión por pares se considera uno de los pilares centrales de la comunicación académica. Aunque existen diferentes modalidades, todas comparten el mismo principio fundamental: garantizar la integridad, el rigor y la fiabilidad de la producción científica^2^.

En el modelo de revisión posterior a la publicación, los manuscritos se someten a una evaluación técnica básica (que abarca el plagio, la ética y el formato) y se publican inmediatamente en plataformas específicas (como F1000Research). La evaluación crítica por parte de la comunidad científica se lleva a cabo de forma transparente en la página del artículo, lo que permite a los autores publicar versiones actualizadas basadas en los comentarios recibidos. El principal desafío radica en la participación; sin la presión editorial de las revistas tradicionales, muchos artículos pueden no recibir comentarios, lo que crea un “vacío de evaluación” para los trabajos menos destacados.

El *Committee on Publication Ethics* (COPE)^3^ ofrece algunas orientaciones para los revisores (traducción libre):

“Los revisores desempeñan un papel importante a la hora de garantizar la integridad del registro académico, pero a menudo asumen esta función sin ninguna orientación y sin ser conscientes de sus obligaciones éticas.

Se espera que los revisores proporcionen una crítica imparcial y constructiva del manuscrito. Las revisiones deben ser objetivas y constructivas, garantizando que los comentarios sean claros y útiles para los autores.

Los revisores deben respetar la confidencialidad de los manuscritos que evalúan. No deben divulgar ninguna información sobre el trabajo ni utilizarla en beneficio propio, garantizando así la integridad del proceso de revisión.

Los revisores deben declarar cualquier posible conflicto de intereses. Esto incluye relaciones financieras, personales o rivalidades académicas que puedan afectar a su imparcialidad.

Se anima a los revisores a que concluyan sus evaluaciones con prontitud para facilitar los procesos de publicación en tiempo oportuno. Los retrasos pueden impedir la divulgación de importantes resultados de investigación y afectar a la carrera de los autores.

Las obligaciones éticas de los revisores incluyen evitar sesgos, respetar los derechos de propiedad intelectual y reconocer las contribuciones de terceros. Los revisores deben mantener los más altos estándares éticos en sus evaluaciones.”

Los revisores son responsables de examinar la precisión, claridad e importancia de un manuscrito, actuando como verdaderos “guardianes” en la difusión del conocimiento científico^4^.

Dado que la transparencia es la base de la integridad del proceso de evaluación, este debe ser abierto, de modo que sea posible identificar a todos los actores involucrados y todas las comunicaciones en el proceso. Hay varias formas de abrirlo.

El SciELO (*Scientific Electronic Library Online*)^5^ recomienda que la revisión por pares de los manuscritos sea lo más informada posible. Los artículos deben contener en su versión final al menos el nombre del editor o editores responsables del proceso de evaluación. Las revistas también deben ofrecer a los revisores y autores la opción de divulgar sus respectivas identidades, con el fin de favorecer la interacción en el proceso de evaluación del manuscrito.

Sin embargo, existen críticas recurrentes al proceso de revisión por pares, entre las que se incluyen retrasos en el ciclo editorial, conflictos de intereses, sesgos conscientes o inconscientes y exceso de trabajo para los revisores. La carga excesiva que soportan los revisores es una realidad ampliamente reconocida: se solicita repetidamente la colaboración de revisores experimentados, quienes aceptan estas tareas voluntarias porque entienden que, sin su participación continua, dejarían de existir las revistas revisadas por pares, lo que retrasaría el progreso de la ciencia.

En esta búsqueda por optimizar el tiempo y atender con éxito todas las demandas impuestas por la ciencia, muchos revisores están utilizando la inteligencia artificial (IA) para la revisión por pares. Inicialmente, su uso se presentó como una tecnología capaz de apoyar a editores y revisores de manera oportuna, pero su uso requiere cautela y reflexión crítica, ya que plantea importantes desafíos. Uno de los principales riesgos es la posibilidad de reforzar los sesgos ya presentes en la literatura científica, ya que estos sistemas se entrenan con datos que pueden reproducir desigualdades y patrones discriminatorios de género, etnia, institución de origen o estilo de redacción. Otro problema radica en la incapacidad de la IA para evaluar sutilezas teóricas, argumentativas y epistemológicas. Mientras que un revisor humano tiene en cuenta el contexto, la originalidad y la relevancia científica, la IA tiende a operar basándose en patrones formales, sin captar matices conceptuales o avances disruptivos. Esto puede dar lugar a evaluaciones injustas, lo que dificulta el desarrollo de nuevas perspectivas^6^. Además, los modelos de IA suelen funcionar como “cajas negras”, sin transparencia sobre los criterios utilizados para sus recomendaciones, algo incompatible con la necesidad de claridad en el proceso editorial científico.

Es muy importante que lo periódico tenga reglas claras en su política editorial sobre si permite o no el uso de IA tanto para autores como para revisores, y que estos sean explícitos sobre qué IA han utilizado en el proceso y cómo. El producto generado siempre debe ser revisado por humanos para corregir cualquier fallo, lo cual es fundamental.

La revisión por pares sigue siendo un dilema para editores y autores. Cuanto más abierto sea el proceso, mayor será la transparencia y mayor será la difusión del conocimiento, ya que el intercambio de comunicación entre el autor, el revisor y el editor permitirá una mayor comprensión del conocimiento generado.

Una de las críticas al sistema es la posibilidad de acoso, intimidación y boicot (exclusión) debido a la divulgación de nombres; sin embargo, incluso la evaluación anónima no garantiza la protección.

Por el contrario, la revisión por pares abierta permite una mayor red de interacción entre el revisor y la academia, además de atribuir el debido crédito por la autoría al revisor, creando un banco de revisiones que puede servir de orientación para otros revisores, especialmente los principiantes.

No sea el último en adherirse.
